# Intravenous fluid therapy in patients with severe acute pancreatitis admitted to the intensive care unit: a narrative review

**DOI:** 10.1186/s13613-022-01072-y

**Published:** 2022-10-17

**Authors:** Andrea Crosignani, Stefano Spina, Francesco Marrazzo, Stefania Cimbanassi, Manu L. N. G. Malbrain, Niels Van Regenmortel, Roberto Fumagalli, Thomas Langer

**Affiliations:** 1 School of Medicine and Surgery, University of Milan-Bicocca, Milan, Italy; 2 Department of Anaesthesia and Critical Care, ASST Grande Ospedale Metropolitano Niguarda, Milan, Italy; 3 General Surgery and Trauma Team, ASST Grande Ospedale Metropolitano Niguarda, Milan, Italy; 4 Department of Pathophysiology and Transplantation, University of Milan, Milan, Italy; 5 First Department of Anaesthesia and Intensive Therapy, Medical University of Lublin, Lublin, Poland; 6 International Fluid Academy, Lovenjoel, Belgium; 7 Department of Intensive Care Medicine, Antwerp University Hospital, Antwerp, Belgium; 8 Department of Intensive Care Medicine, Ziekenhuis Netwerk Antwerpen Campus Stuivenberg, Antwerp, Belgium

**Keywords:** Acute pancreatitis, Critical illness, Fluid therapy, Crystalloid solutions, Ringer’s lactate

## Abstract

Patients with acute pancreatitis (AP) often require ICU admission, especially when signs of multiorgan failure are present, a condition that defines AP as *severe*. This disease is characterized by a massive pancreatic release of pro-inflammatory cytokines that causes a systemic inflammatory response syndrome and a profound intravascular fluid loss. This leads to a mixed hypovolemic and distributive shock and ultimately to multiorgan failure. Aggressive fluid resuscitation is traditionally considered the mainstay treatment of AP. In fact, all available guidelines underline the importance of fluid therapy, particularly in the first 24–48 h after disease onset. However, there is currently no consensus neither about the type, nor about the optimal fluid rate, total volume, or goal of fluid administration. In general, a starting fluid rate of 5–10 ml/kg/h of Ringer’s lactate solution for the first 24 h has been recommended. Fluid administration should be aggressive in the first hours, and continued only for the appropriate time frame, being usually discontinued, or significantly reduced after the first 24–48 h after admission. Close clinical and hemodynamic monitoring along with the definition of clear resuscitation goals are fundamental. Generally accepted targets are urinary output, reversal of tachycardia and hypotension, and improvement of laboratory markers. However, the usefulness of different endpoints to guide fluid therapy is highly debated. The importance of close monitoring of fluid infusion and balance is acknowledged by most available guidelines to avoid the deleterious effect of fluid overload. Fluid therapy should be carefully tailored in patients with severe AP, as for other conditions frequently managed in the ICU requiring large fluid amounts, such as septic shock and burn injury. A combination of both noninvasive clinical and invasive hemodynamic parameters, and laboratory markers should guide clinicians in the early phase of severe AP to meet organ perfusion requirements with the proper administration of fluids while avoiding fluid overload. In this narrative review the most recent evidence about fluid therapy in severe AP is discussed and an operative algorithm for fluid administration based on an individualized approach is proposed.

## Introduction

Acute pancreatitis (AP) is an acute inflammatory disease of the pancreas and is among the most common gastrointestinal disorders requiring hospitalization. About 80% of patients with AP have a mild, self-limiting form that needs only brief treatment in a non-critical setting. However, 15–20% of AP episodes are moderately severe or severe, potentially leading to multi-organ failure (MOF), and are burdened by a 20–40% mortality rate [1].

The Atlanta classification for pancreatitis considers two types of AP (interstitial and edematous/necrotizing) and a 3-grade severity scale (mild, moderately severe, severe). Interstitial pancreatitis refers to a diffuse inflammatory edema, while necrotizing pancreatitis is characterized by necrosis involving pancreatic parenchyma and/or the peripancreatic tissue. Both interstitial and necrotizing pancreatitis can be severe; however, interstitial pancreatitis is usually of mild severity, commonly improves in 48 h, and has a mortality rate below 5%. On the other hand, necrotizing pancreatitis, which is observed in 5–10% of patients with AP, often shows a more severe course [2, 3]. The severity of AP is strictly related to the development of MOF, usually involving the cardiocirculatory, renal and pulmonary systems. If organ failure lasts less than 48 h, AP is defined as moderately severe, while it is defined as severe (severe acute pancreatitis, SAP), if organ failure persists for more than 48 h [4]. The discussion of complications not in direct relation with MOF (e.g., pancreatic pseudocysts and perforation of hollow viscus) is beyond the scope of the review and can be found elsewhere [5].

Two peaks of mortality have been identified, i.e., (*I*) during and (*II*) after the first week from symptoms development (“early” and “late” phase, respectively) [2]. The early phase is usually characterized by a sterile inflammatory process starting from the pancreas and progressing to a systemic level [6–8]. In this phase, organ failure is linked to the systemic inflammatory response syndrome (SIRS) and has a mortality rate of up to 50%, causing about half of all deaths due to SAP. The second peak of mortality occurs in the “late phase” and is usually secondary to infections of the pancreatic necrotic debris [2, 6, 9–11].

Intensive care unit (ICU) admission is warranted in case of organ failure. With the exception of some special situations (e.g*.*, endoscopic retrograde cholangiopancreatography), no specific therapies exist. The treatment of SAP is, therefore, supportive, mainly based on fluid administration, enteral nutrition, and pain management [12]. Many authors consider intravenous (IV) fluid therapy as the treatment cornerstone for SAP, especially during the first 24 h after disease onset [13].

The aim of this narrative review is to discuss the most recent evidence about fluid therapy in severe AP and to propose an operative algorithm based on an individualized approach to administering intravenous fluids with respect to clinical, hemodynamic, and laboratory monitoring.

## Hypovolemia and organ failure

### Pathophysiology

**Fig. 1:**
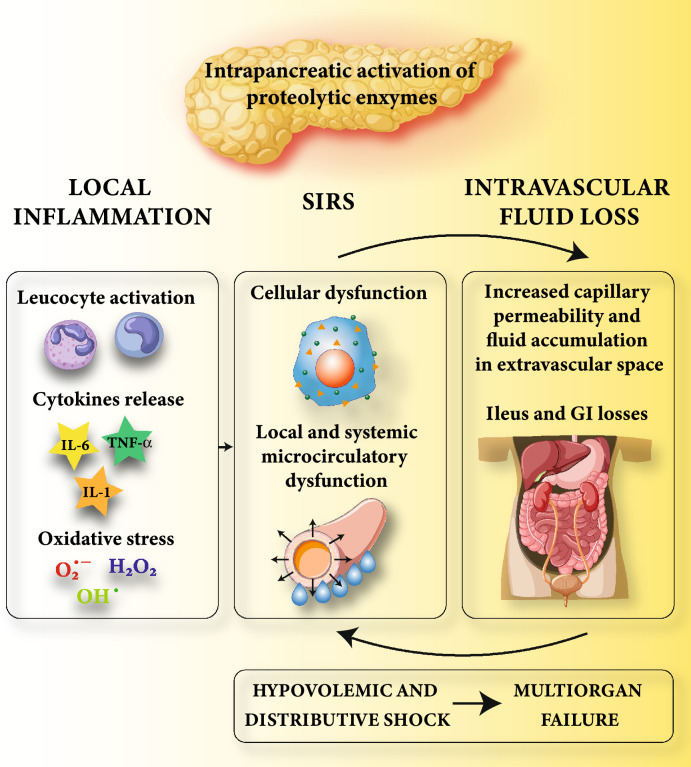
Pathophysiology of fluid loss/hypovolemia in patients with severe acute pancreatitis. The intrapancreatic activation of proteolytic enzymes causes local tissue inflammation with leukocyte activation, cytokines, and reactive oxygen species release. At a systemic level, the massive release of pro-inflammatory cytokines from the injured pancreas leads to SIRS, causing an intravascular fluid loss through vasodilatation, cellular dysfunction, and increased capillary permeability. Ileus, vomit, decreased fluid intake, and increased insensible losses further contribute to intravascular fluid depletion. If not interrupted, this vicious cycle leads to a severe hypovolemic and distributive shock and ultimately to MOF. *IL-1* Interleukin-1; *TNF-α* Tumor necrosis factor; *IL-6* Interleukin-6; *SIRS* Systemic inflammatory response syndrome; *GI* Gastrointestinal; *MOF* Multiorgan failure

Fluid loss and cytokine release are the two main pathological mechanisms that contribute to AP severity. On the one hand, a massive pancreatic release of pro-inflammatory cytokines (IL-1, TNF-α, and IL-6) leads to SIRS, vasodilatation, and cellular dysfunction [7]. On the other hand, several factors contribute to extensive intravascular fluid loss (Fig. 1). First, vomit and abdominal pain impair feeding and enhance gastrointestinal fluid depletion. Second, insensible losses are increased by the tachypnea and diaphoresis related to pain, systemic inflammation, and fever. Finally, and most importantly, pancreatic inflammation and SIRS are associated with local and systemic increased capillary permeability, favoring extravascular fluid accumulation [7, 14, 15]. Of note, local microcirculatory dysfunction contributes to pancreatic tissue hypoperfusion and ischemia [14, 16] which can spread systemically and further worsen the pancreatic insult [17]. This vicious cycle might be deleterious also for the hollow viscera [18]. In particular, ileus and fluid accumulation within the intestinal lumen might occur. In addition, fluid sequestration frequently occurs in the retroperitoneal space [19], potentially causing intra-abdominal hypertension and abdominal compartment syndrome [20–22].

In summary, patients with SAP are frequently characterized by a severe hypovolemic and distributive shock, which ultimately leads to the development of MOF [23–25]. The extent of fluid depletion/shifts is, however, difficult to assess precisely. Up to four liters of fluid sequestration have been reported in mild pancreatitis at 48 h, and up to six liters in more severe forms [26, 27]. In several studies, greater fluid sequestration has been associated with a higher complication rate and morbidity [28–31].

## Fluid resuscitation: general recommendations

Available guidelines for the treatment of AP underline the importance of early fluid therapy [13, 32–37]. However, there is currently no consensus neither about the type nor about the optimal fluid rate or goal of fluid administration [38, 39] (Table 1). In this context, three main features of AP should be kept in mind. First, AP is a dynamic illness that can worsen after initial presentation [40, 41]. Continuous monitoring and reassessment of fluid requirements are, therefore, warranted. Second, AP is considered a time-dependent illness whose outcome could be influenced by the promptness of interventions. Indeed, the first hours from disease onset are considered pivotal to prevent progression of SIRS, MOF, and/or worsening of pancreatic necrosis [42]. On the other hand, however, the administration of excessive amounts of fluids might be detrimental in the presence of an increased capillary-permeability state [6, 43, 44]. Third, hypovolemia in the context of SAP is not a simple loss of intravascular volume, but a combination of hypovolemia and microcirculatory dysfunction due to SIRS. Of note, it is conceivable that fluid therapy might play a role in modulating inflammation and reducing capillary permeability [44–47].

**Table 1:** Main studies investigating fluid administration in acute pancreatitis

Authors	Study design	Type of IV fluid	Rate of administration	Resuscitation endpoint	Study endpoint	Results
De-Madaria E, [130]	RCT (*n* = 249)	RL	*Aggressive*: bolus 20 ml/kg–3 ml/kg/h *Moderate*: bolus 10 ml/kg–1.5 ml/kg/h. In both groups, assessments at 3, 12, 24, 48, and 72 h to check for fluid overload or hypovolemia	BUN, Ht, UO, signs of dehydration, SBP	Development of moderately severe or severe pancreatitis during the hospitalization. Safety outcome signs of fluid overload	No difference in the primary outcome between the two groups. Higher incidence of fluid overload in the aggressive resuscitation group
Gad MM, [39]	Metanalysis (*n* = 2686)	/	*Aggressive* vs *Non-aggressive*	/	Mortality, PN, OF, AKI, RF	No difference between the two groups
Li L, [134]	RC (*n* = 912)	/	*Rapid*: ≥ 3 ml/kg/h *Slow*: < 3 ml/kg/h	/	Rate of MV, LOS	MV and hospital LOS associated with rapid FT in SAP and Ht ≥ 44%
Cuèllar-Monterrubio JE, [135]	RCT (*n* = 88)	Hartmann solution	*Aggressive*: bolus 20 ml/kg–3 ml/kg/h for 24 h -30 ml/kg/day *Non-aggressive*: 1.5 ml/kg/h for 24 h, then 30 ml/kg/day	Vital signs, UO, BUN, Ht, lactate, SIRS	Development of SIRS and OF	No difference in outcome
Ye B, [88]	RC (*n* = 179)	NS, RL	*Aggressive* (> 4 L/day) *Non-aggressive* (< 4 L/day)	Vital sign, UO > 0.5 ml/kg/h, Ht < 45%	AKI development Chloride exposure	> 4 L/day and higher chloride exposure associated with AKI
Yamashita T, [51]	RC (*n* = 1097)	/	*Aggressive* ≥ 6 lt/day *Non-aggressive* < 6 lt/day	/	In-hospital mortality	≥ 6 L within 24 h associated with less mortality
Buxbaum JL, [131]	RCT (*n* = 60)	RL	*Aggressive*: 20 ml/h bolus + 3 ml/kg/h *Standard*: 10 ml/h + 1.5 ml/kg/h (median 3.91 L/24 h)	Ht, BUN, Cr	Decrease in Ht, BUN, Cr Reduced pain Tolerance to oral feeding	Higher clinical improvement, reduced SIRS development, and less hemoconcentration in *Aggressive* group
Singh VK, [77]	RC (*n* = 1010)	/	*FVER* Group I < 500 ml Group II 500–1000 ml Group III > 1000 ml *FV24* Group I < 3200 ml Group II 3200 – 4300 ml Group III > 4300 ml	/	LC, OF, Invasive treatment, mortality	FVER 500–1000 ml and > 1000 ml associated with better outcomes FV24 > 4300 ml associated with higher LC
Weitz [136]	RC (*n* = 391)	Ringer’s solution	/	/	Severity, LC, OF, PN	Higher fluid volume associated with severity and LC
Wall I, [50]	RC (*n* = 286)	/	Until year 1998 = 113 ml/h in first 6 h From 1998 to 2008 = 284 ml/h in first 6 h	/	OF, PN, mortality	Less mortality and PN up to year 2008
Warndorf MG, [49]	RC (*n* = 434)	NS (in 85% of cases)	*Early FT*: ≥ 1/3 of the total 72 h fluid volume administered in the first 24 h *Late FT:* < 1/3 administered in the first 24 h (2.403 ml/24 h)	/	Mortality, SIRS, OF, ICU, LOS	Less SIRS, ICU, OF associated with early FT
De-Madaria E, [74]	RC (*n* = 247)	NS *plus* D5%/D10%	FT volume in first 24 h: Group A: < 3.1 L Group B: 3.1 – 4.1 L Group C: > 4.1 L	Ht < 44%, UO > 50 ml/h, low Cr, normal SBP	OF, PN, APFC, mortality	Group C had more RF and AKI rate
Kuwabara K, [76]	RC (*n* = 9849)	Crystalloids	*FV48* *FVR*	/	Mortality, MV, Dialysis	Higher FV48 associated with *increased* rate of MV, dialysis, mortality Higher FVR associated with *lower* mortality in the severe AP group
Wu B, [47]	RCT	NS vs RL	Standard 20 ml/kg bolus + 3 ml/kg/h vs physician judgment	BUN	SIRS	No difference between different rates; difference between RL and NS
Mole DJ, [137]	RC (*n* = 30)	NS, HS, D5-50%, sodium bicarbonate, phosphate; colloids (Gelofusine, Albumin 4.5%); blood products	/	Physician’s judgment	Volume of fluids administered	Less fluids associated with higher mortality
Gardner TB, [48]	RC (*n* = 45)	NS (71%), D5% + NaCl 0.45% (20%), RL (9%)	*Early FT*: 203 ml/h in first 24 h *Late FT:* 71 ml/h in first 24 h	/	Mortality, OF, LOS	Higher mortality rate in Agg group
Mao E, [79]	RCT (*n* = 115)	NS, RL, plasma, HES 6%	Depending on goal-Ht	Ht < 35% vs > 35%	Incidence of sepsis, mortality	Goal Ht < 35%: major incidence of sepsis and higher mortality rate. Higher amount of fluid volume
Mao E, [78]	RCT (*n* = 76)	NS, RL, plasma, HES 6%	Group I: 10–15 ml/kg/h Group II: 5–10 ml/kg/h	HR, MAP, UO, Ht < 35%	APACHE II score, MV, ACS and sepsis incidence, mortality	Group I: higher incidence of MV and ACS, higher mortality rate
Eckerwall G [75]	RC (*n* = 99)	Crystalloids, Colloids (mainly albumin)	> 4000 ml/24 h Vs< 4000 ml/24 h	/	Respiratory complications, ICU admission rate, mortality	More respiratory complications and need for intensive care admissions with > 4000 ml/24 h

/ Not specified; *AKI* Acute kidney injury; *APFC* Acute peripancreatic fluid collections; *ACS* Acute Compartment Syndrome; *BUN* Blood urea nitrogen; *Cr* Creatinine; *D*5–10–50% Dextrose solution 5–10–50%; *FT* fluid therapy; *FVER* Fluid Volume in Emergency Room, within 4 h from admission; *FV24* Fluid volume administered in first 24 h, since admission to the hospital ward. *FV48* Fluid volume per day in the initial 48 h; *FVR* (Fluid volume ratio) Average fluid volume per day in the first 48 h, compared to fluid volume per day during total hospitalization; *HD* Hemodialysis; *Ht* Hematocrit; *HES* 6% Hydroxyethyl starch 6%; *ICU* Intensive Care Unit; *LOS* Hospital Length of stay; *LC* Local complications; *MV* Mechanical ventilation; *NS* Normal saline; *PN* Pancreatic necrosis; *OF* organ failure; *RF* Respiratory failure; *RC* Retrospective Cohort, *RCT* Randomized Clinical Trial; *RL* Ringer Lactate; *SBP* Systolic blood pressure; *SIRS* Systemic Inflammatory Response Syndrome; *UO* Urinary output

Some studies have shown that the rapid reversal of hypovolemia is associated with improved outcomes [48–52]. In addition, multiple animal experiments suggested that adequate fluid therapy could reduce pancreatic damage and in some cases mortality [53–55]. Based on these results, most guidelines advocate for early and adequate fluid resuscitation.

Some laboratory values such as hematocrit (Ht) and blood urea nitrogen (BUN) have been traditionally considered markers of hypovolemia and might contribute to the assessment of fluid status. High values at admission and their increase during the first 24–48 h could thus indicate inadequate fluid resuscitation [56, 57].

Hemoconcentration (i.e*.*, high Ht) is associated with high fluid sequestration and increased blood viscosity, which, by itself, might contribute to impaired pancreatic microcirculation, favoring pancreatic necrosis [28]. Several studies reported a higher probability of severe disease when Ht ≥ 45% at admission [57–60]. Likewise, the failure to reduce Ht within the first 24 h has been linked to inadequate fluid therapy and worse outcomes.

In addition, hypovolemia might lead to an increase in BUN. Its specificity is, however, low, as the increase might be multifactorial: hypovolemia, renal failure, increased protein catabolism, and gastrointestinal bleeding [61]. Nevertheless, high BUN values at admission (≥ 20 mg/dL, equivalent to plasma urea ≥ 42 mg/dL), regardless of the underlying cause, and increasing values at 24 h have been found to be predictive of organ failure/mortality [61–65].

While both parameters are discussed in literature, Ht might be a more appealing endpoint to guide fluid resuscitation, as compared to BUN, being the latter more influenced by other pathological conditions that are common in the critically ill patients. Moreover, Ht is relatively simple to measure, as it is commonly calculated from the results of the point-of-care arterial blood gas analysis.

### Infusion rate and cumulative administered volume

The optimal timing and rate of fluid administration are still unknown. Available guidelines recommend *early* and *aggressive* fluid therapy [13, 38]. This definition refers to a *higher* fluid rate in the first hours of the disease and a *lower* rate in the following days.

Gardner et al. retrospectively analyzed fluid administration over the first 72 h of hospitalization in a group of 45 patients with SAP [48]. When more than one-third of the cumulative fluid volume was infused within the first 24 h, the treatment was classified as “*early”,* while it was defined as “*late resuscitation”* if less than one-third of the cumulative volume was infused within the first day. As expected, the difference in fluid volume administered in the first 24 h was marked: almost five liters in the “*early*” and less than 2 liters in the “*late”* resuscitation group. Interestingly, the “*early*” experienced significantly lower mortality as compared to the “*late*” group. Based on Gardner’s and other similar findings, progressively larger amounts of fluid have been administered in the early phases of SAP [49, 50].

Currently, different guidelines suggest a starting fluid rate for patients with AP presenting with features of hypovolemia (Table 2) [13, 33, 34]:

- 5–10 ml/kg/h for the first 24 h until resuscitation goals are achieved [33]. Suggested goals are heart rate (HR) < 120 bpm, mean arterial pressure (MAP) > 65 mmHg, urinary output (UO) > 0.5 ml/kg/h, and Ht 35–44%.
- 250–500 ml/h of isotonic crystalloid for the first 12–24 h, with little benefit beyond this time period and with the goal to decrease BUN and Ht [13].
- 150–600 ml/h in patients with shock or dehydration, until MAP > 65 mmHg and UO > 0.5 ml/kg/h, and 130–150 ml/h in patients without severe signs of hypovolemia [34].

**Table 2:** Suggested fluid therapy regimens in severe acute pancreatitis

Authors, year	IV infusion rate (in the first 24 h)	Goals/endpoints	Comments
De Waele E et al. [70]	5–10 ml/kg/h	/	Up to 250–500 ml/h for 24 h. Up to ≥ 5000 ml may be necessary
Working group IAP/APA*,* [33]; Hines OJ, Pandol SJ*,* [5]	5–10 ml/kg/h	Clinical targets (UO > 0.5 -1 ml/kg/h) Invasive targets (ITBV, SVV) Laboratory markers (Ht 35–44%)	2500–4000 mL in the first 24 h are usually sufficient
Buxbaum et al. [131]	20 mL/kg bolus, then 3 ml/kg/h	Urea, Ht, creatinine	Higher clinical improvement with aggressive IV hydration Tested only on *mild* AP
DiMagno MJ, [66]	5–10 ml/kg/h until hemodynamic stability, then 3 ml/kg/h	HR < 120, MAP 65–86 mmHg, UO > 50 ml/h	After 6 h check BUN: ••••••If < 20 mg/dl or falling: change to 1.5 ml/kg/h ••••••If not, infusion of 5–10 ml/kg/h
Yokoe M et al. [34]	150–600 ml/h	MAP > 65 mmHg and UO > 0.5 ml/kg/h	Reduce to 130–150 ml/h when dehydration and shock are reversed
Pezzilli R et al. [36]	Initial bolus of 20 ml/kg within 30–45 min, then 2 ml/kg/h	Normal UO, MAP, HR. BUN < 20 mg/dL, Ht 35–44%	Monitor every 8–12 h for the first 24–48 h
Aggarwal et al. [67]	Bolus 1000 mL in 1 h, then 3 ml/kg/h (200 ml/h)	UO > 0.5 ml/kg/h, Ht 25–35%, drop in BUN	Continue for 24–48 h, until signs of volume depletion disappear
Tenner S et al. [13]	250–500 ml/h	Decrease Ht and BUN	Benefits are limited to first 12–24 h
Fisher MJ, Gardner TB*,* [82]	250–300 ml/h	Enough to produce a UO of 0.5 ml/kg/h	Tailor on patients’ characteristic, urine output, blood pressure, and modest decrease in hematocrit
Nasr JY, Papachristou GI, [132]	Initial bolus 20 ml/kg, followed by 150–300 ml/h (3 ml/kg/h)	BUN, Ht	Subsequent maintenance: 2–3 ml/kg/h
Wu BU et al. [47]	Bolus 20 ml/kg in 30 min, then 3 ml/kg/h maintenance (1.5 ml/kg/h for less hypovolemic patients)	Decreased BUN level	No improved outcome in early goal directed therapy was evidenced
Pandol S et al. [133]	Level of dehydration: fluid rate -Severe: 500–1000 ml/h -Moderate: 300–500 ml/h -Mild: 250–350 ml/h	Vital signs, UO, Ht	Reassess every 1–2 h

*AP* Acute Pancreatitis; *BUN* Blood urea nitrogen; *HR* Heart rate; *Ht* Hematocrit; *ITBV* Intrathoracic blood volume; *MAP* Mean arterial pressure; *UO* Urine output; *SVV* Stroke volume variation; / Not specified

Other infusion strategies are reported in Table 2. After the early critical phase, fluid rate is usually reduced to 2–3 ml/kg/h [36, 47, 66, 67]. Close clinical monitoring and the definition of clear resuscitation goals are fundamental [68]. Generally accepted targets are urinary output, reversal of tachycardia and hypotension, and improvement of laboratory markers, such as BUN and Ht. The usefulness of different endpoints to guide fluid therapy is still debated. There is, however, general agreement regarding the importance of close monitoring of fluid status, to reduce the risks of fluid overload [13, 69].

In most cases, a cumulative volume of 2.5–4 L during the first 24 h has been shown to be sufficient to reach the resuscitative targets [33]. However, clinicians should be aware that up to five or more liters per day may be required in the initial phase [51, 70].

## Complications of excessive fluid administration

Despite the clear benefits of IV fluid therapy, excessive administration of fluids can lead to several complications. Overall, markedly positive fluid balances are associated with worse outcomes in critically ill patients [71]. Fluid might be retained in the interstitial space leading to interstitial edema, impaired organ perfusion, and possibly acute pulmonary edema. In the specific context of SAP, intestinal wall edema and retroperitoneal edema are feared complications for abdominal compartment syndrome development [22]. A global increased permeability syndrome (GIPS) might develop in the context of persistent systemic inflammation (i.e*.*, high capillary leak) and positive cumulative fluid balance (i.e*.*, edema formation and polycompartment syndromes) with persistent organ failure [72, 73]. It is, therefore, fundamental to tailor fluid therapy carefully.

Many authors described in observational studies an association between high IV fluid volumes and increased intra-abdominal pressure, increased organ failure, and mortality in the specific setting of AP [74–76]. The observational nature of these studies, despite multiple corrections and normalizations, limits the soundness of the findings, as patients with more severe forms of AP usually require more fluids and have a worse prognosis. In this context, it is, therefore, difficult to establish the definitive causal relationship between fluid volume and outcome [43, 77].

To date, only a few randomized clinical trials (RCTs) have compared a more aggressive versus a more conservative resuscitation strategy. A Chinese group conducted two RCTs on patients with SAP. In the first study [78], a group received a fixed fluid rate of 10–15 ml/kg/h, while the second group received 5–10 ml/kg/h, as needed to achieve hemodynamic stability. In the second trial [79], a group received IV fluids at admission rapidly aiming at a Ht < 35%, while the other group had a Ht target > 35%. In both studies, the authors report a higher incidence of sepsis, higher complications due to fluid overload, and a higher mortality rate when fluids were administered in high volume. The results of these studies are, however, not definitive, as several flaws (unclear randomization method, unreported incidence of necrosis, contradictory data on the amount of volume infused) have been identified [80–82].

In summary, it is still unknown how to identify the correct amount and rate of IV fluid to prevent or reverse the evolution of organ failure and reduce the complications due to fluid overload.

According to the most recent evidence and experts’ opinion, fluid therapy should be tailored based on patient’s needs, enhanced in the first hours, and continued only for the appropriate time frame. For this reason, fluid therapy is usually discontinued or significantly reduced after the first 24 after admission. Of note, patients who do not show a prompt clinical response after the first 6–12 h of fluid therapy might not benefit from a large fluid administration [13, 66, 82, 83].

## Types of intravenous fluids

The ideal IV fluid in the context of SAP should improve hemodynamics/organ perfusion by restoring extracellular fluid volume while modulating inflammatory response in the presence of altered capillary permeability. Crystalloids and colloids are the two broad fluid categories available in the critical care setting. In theory, they have different distributions within fluid compartments, resulting in different intravascular volume expansions. Importantly, they show different adverse effects [84–86].

Studies comparing different fluid administration strategies in AP used different types or combinations of IV fluids: normal saline (NS), balanced crystalloids, or mixed strategies using a combination of crystalloids and colloids (such as albumin, starches, and fresh frozen plasma) [38]. Different IV fluids likely have a different impact on some clinical outcomes [87]. In a retrospective study, Ye et al. observed that an aggressive resuscitation strategy was associated with an increased incidence of acute kidney injury in patients with SAP [88]. Of note, both high volume resuscitation (> 4 L in the first 24 h) and high chloride exposure due to NS infusion were independent risk factors for acute kidney injury. High concentrations of serum chloride have been associated with renal failure in critically ill patients also in other studies [87, 89].

### Crystalloids

Normal saline (0.9% sodium chloride solution) and balanced crystalloid solutions (such as Ringer’s lactate) are broadly used fluids. Balanced solutions are more similar to extracellular fluid as they contain some organic anions (buffers), which are metabolized once delivered to the patient and allow to lower the chloride concentration of the fluid. Different crystalloids have different strong ion differences (SID), i.e*.*, the difference between strong cations (mainly [Na +]) and strong anions ([Cl-]). According to Stewart’s acid–base approach, SID is an independent variable affecting the pH of a biological solution [90, 91]. Normal plasma SID ranges between 33–40 mEq/L, according to the used definition. A reduction in SID, shifts the system toward acidosis while an increase in SID toward alkalosis [92]. The SID of infused crystalloids (after metabolism of the organic anions) might, therefore, significantly alter plasma SID and, therefore, affect pH [85, 86]. Normal saline has a SID of 0 mEq/L as Na^+^ and Cl^−^ have the same concentration, and its net effect is, therefore, always acidifying. On the other hand, the infusional SID of balanced crystalloids ranges between 28 and 55 mEq/L with a reduced effect on plasma acid–base [93].

Ringer’s lactate solution (RL), a slightly hypotonic, balanced crystalloid, has been compared to NaCl 0.9% for fluid resuscitation in a small RCT in patients with AP [47]. In this trial, patients randomized to RL had a reduced prevalence of SIRS and a lower concentration of C-reactive protein at 24 h post-admission. Similar results were described by other authors [46, 94–97].

These findings might be explained by a possible immunomodulatory, anti-inflammatory, and organ-protective effect of lactate, but also to a detrimental effect of high chloride concentrations [98]. Indeed, experimental animal studies suggest that an exogenous hydrochloric acid load might worsen AP, as the local acidification at the pancreatic acinar level could favor pancreatic edema/necrosis [99]. While clinical data on this topic are lacking, these findings provide an additional rationale for avoiding the exogenous acid load resulting from the infusion of large volumes of NaCl 0.9%. Finally, it might be worth mentioning that isotonic fluids, besides providing water, contain large quantities of sodium, which might contribute to water and salt overload, ultimately favoring edema formation [100–102].

### Colloids

Colloids are solutions based on semi-synthetic or plasma-derived molecules dissolved in crystalloids. Human albumin, starches, gelatines, and dextrans are the colloids used in clinical practice. In theory, these molecules are large enough to be retained by semi-permeable membranes and should exert higher colloidal-osmotic pressures than crystalloids. Their putative advantage is the achievement of higher volume expansions with less infused volume and the longer persistence in the intravascular space, conceptually leading to less edema formation and better hemodynamic stability [71, 84]. However, their use in critically ill patients is highly debated [89]. Indeed, no definite benefit exists over crystalloids on mortality, and colloids are rather known for their potentially harmful effects [103].

In the specific context of AP, experiments conducted in animals suggest that resuscitation with dextrans could be superior to crystalloids, possibly due to improved pancreatic perfusion [104, 105]. In humans, only a few studies assessed the use of colloids for fluid resuscitation of patients with AP/SAP. Zhao et al. compared patients treated with hydroxyethyl starch (HES) and NS in a 1:1 ratio to patients treated with NS alone and observed a shorter time to hemodynamic stability and microcirculation perfusion improvement in the first group [45]. However, HES administration has been largely investigated in critically ill patients and the adverse effects have been shown in large RCTs [106, 107]. Thus, current evidence does not support HES use in most patients admitted to the ICU, including those with SAP [108].

It is, therefore, clear that no conclusive evidence exists about the ideal fluid in SAP and guidelines do not provide a definitive indication, given the moderate quality of the available evidence [109, 110]. However, most experts and guidelines recommend crystalloids, and among them, RL is usually indicated as the fluid of choice [111].

## Fluid resuscitation: an individualized approach

Recent data support the importance of a tailored and individualized fluid therapy in the context of SAP requiring ICU admission [112]. As described above, early and aggressive fluid therapy can be beneficial for some, but deleterious for other patients. Aggressive fluid therapy might be well-tolerated in patients with mild AP, as the patient is able to eliminate fluids in excess. On the contrary, patients with SAP have persistent organ failure and markedly increased vascular permeability. Here, a large amount of IV fluids might lead to water and salt overload and further worsen the disease [68, 83, 113]. Given the potential harm of an inappropriate administration of fluids in critically ill patients, patients need to be carefully monitored [114].

Clinical endpoints that could guide fluid resuscitation in patients with SAP can be classified in three groups: noninvasive clinical parameters (1), invasive hemodynamic parameters (2), and laboratory markers (3).

1. Clinical parameters that are commonly and easily monitored are MAP, HR, and UO. A high HR and/or a low MAP and UO can be indicative of low circulating blood volume, oxygen delivery, and impaired end-organ perfusion [115]. An HR < 120/min, a MAP between 65 and 85 mmHg, and UO > 0.5 ml/kg/h are desirable endpoints in the management of SAP. Abnormal values alone or with other signs of organ hypoperfusion should prompt fluid administration in the early phase, [116, 117]. The skin mottling score and the capillary refill time are other useful clinical markers of microvascular perfusion and could be helpful clinical parameters in this context [118].
2. Invasive hemodynamic parameters, such as those obtained from a central line catheter or from the arterial pulse contour analysis, are useful to assess hypovolemia and fluid responsiveness. Static indices of cardiac preload like central venous pressure (CVP) are still used in common practice, though their use is highly debated. CVP values might be useful as a safety limit to avoid fluid overload in the setting of right-heart failure. Calibrated hemodynamic monitoring systems, such as PiCCO (Pulsion Medical Systems SE, Feldkirchen, Germany), are based on transpulmonary thermodilution. Such systems are commonly available in the ICU and have been investigated also in the specific setting of SAP with promising results [119–122], showing that PiCCO parameters could better correlate with changes in cardiac output and could guide fluid resuscitation with favorable outcomes.
3. Lactate levels and central venous saturation are indirect markers of organ perfusion and oxygen delivery [123]. Inadequate organ perfusion and inadequate oxygen utilization at the cellular level are the ultimate result of massive fluid loss in early SAP and also a key feature of hemodynamic shock state. In addition, Ht and BUN, as previously mentioned, are useful laboratory markers which could help estimate the degree of fluid sequestration at admission.

Four distinct phases of fluid resuscitation for critically ill patients have been recently proposed and conceptualized through the R.O.S.E. acronym (Resuscitation, Optimization, Stabilization, Evacuation), which could also be adapted for patients with SAP [114]. In the hyperacute phase (phase 1, *Resuscitation*), the patient might be in hypovolemic shock and the physician must provide an early, adequate goal-directed fluid management with an abundant fluid infusion. A positive fluid balance is inevitable and tolerated to achieve adequate perfusion. In the *Optimization* phase (phase 2), although still hypovolemic, patients with SAP present a more compensated shock. The individual fluid requirement must be regularly assessed. The goal is to maintain adequate tissue oxygenation to limit organ damage and to maintain a neutral fluid balance to avoid fluid overload. Intra-abdominal pressure measurement has been advocated in patients with abdominal problems, to monitor the possible development of abdominal compartment syndrome [124, 125]. The *Stabilization* (phase 3) evolves over the following days and signs of circulatory shock are absent. Fluids are needed only to replenish ongoing losses. Finally, the *Evacuation* (phase 4) starts with spontaneous evacuation (the “flow” phase) when the acute insult resolves. When evacuation is not spontaneous, a strategy of active fluid removal, using diuretics, might be pursued [71]. If this model is applied to the clinical context of SAP in its early phase, most of the fluid therapy is directed to reverse hypovolemia in the *Resuscitation* and *Optimization* phases. In both cases, an individualized approach for fluids administration is advocated.

**Fig. 2:**
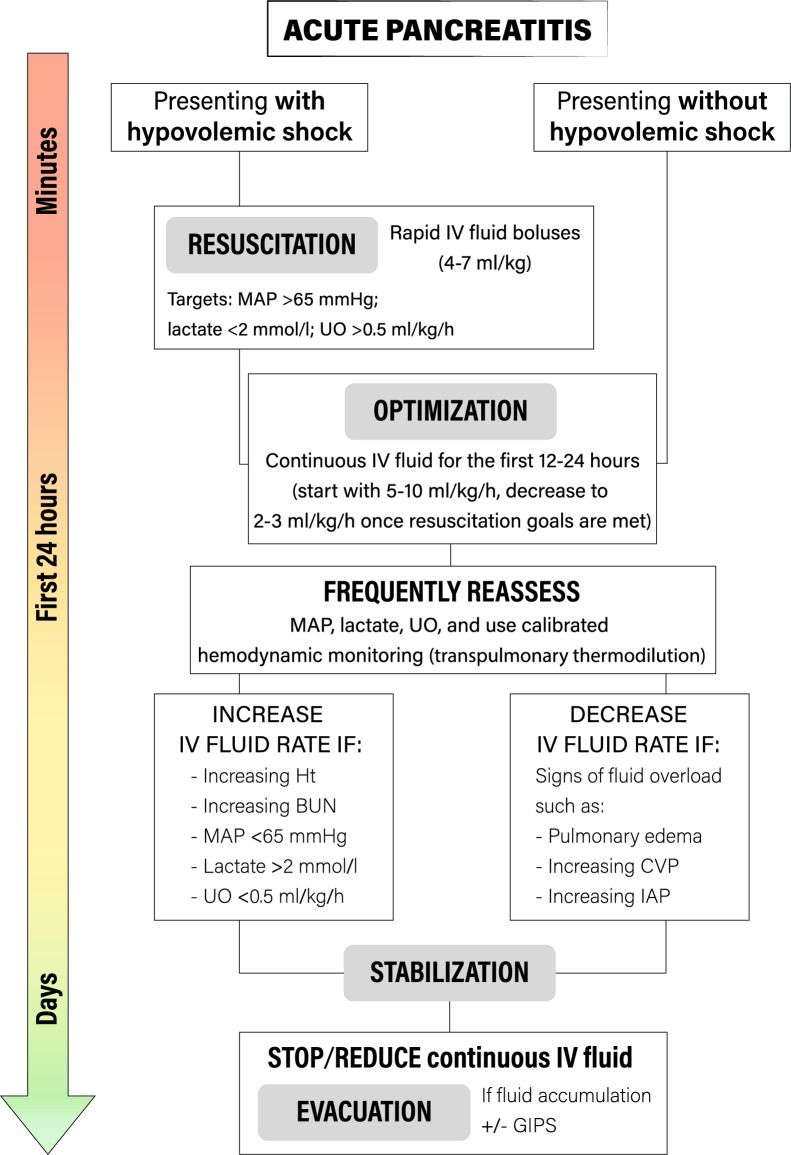
Proposed algorithm for fluid resuscitation in severe acute pancreatitis. Hypovolemic shock is reversed with intravenous balanced crystalloids until *Resuscitation* goals are met. In the *Optimization* phase, a continuous infusion should be provided to meet ongoing fluid losses. A continuous *Reassessment* is required to assess further needs for fluids, guided by advanced hemodynamic monitoring systems, aiming to define the real fluid requirements while evaluating any signs of fluid overload. The *Stabilization* evolves over the following days. Here, fluids are needed only to replenish ongoing losses and the *evacuation* starts with spontaneous or induced evacuation when the acute insult resolves. *IV* Intravenous; *MAP* Mean arterial pressure; *UO* Urinary output; *Ht* Hematocrit; *BUN* Blood urea nitrogen; *IAP* Intra-abdominal pressure; *CVP* Central venous pressure; *GIPS* Global increased permeability syndrome

A single clinical marker alone unlikely reflects the overall volume status and the assessment of multiple parameters simultaneously is considered more reliable [113]. The patient should be frequently reassessed during the first 24 h, ideally every 2–3 h to adjust fluid administration based on modification in these parameters, to avoid under- or over-treatment [56, 68] (Fig. 2).

Over the first Resuscitation phase and the following Optimization phase, the use of vasopressors (like norepinephrine) could be considered [126]. During SAP, hypotension is partially due to vasodilation and an hyperdynamic circulatory state usually follows fluid resuscitation [127, 128]. In the very early phase, fluids are virtually mandatory, but vasopressors might be administered as an adjunct to temporarily increase a low MAP—while fluid resuscitation is ongoing. During the following stages, fluid requirement is constantly reassessed: a vasopressor can be used when a low MAP is a concern, but the patient seems otherwise euvolemic. However, a patient with SAP and clear signs of hypovolemia should not receive vasopressor instead of fluids, given the risk of further organ hypoperfusion. Once fluid status is optimized de-escalation should be considered [129].

## Recent and ongoing research

Three main questions are still not completely answered about fluid therapy during SAP. First, the optimal rate and the extent of fluid administration in the early phase of the disease. Second, the most accurate clinical and laboratory endpoint to guide fluid resuscitation. Third, the best fluid that, administered in large volume, could guarantee the highest efficacy and safety [38].

WATERFALL is a very recently published RCT [130]. The study aimed at comparing aggressive versus moderate fluid resuscitation in patients with AP. Patients who met the criteria for moderately severe or severe disease at baseline were excluded. A total of 122 subjects received an aggressive (RL 20 ml/kg bolus administered over 2 h followed by RL 3 ml/kg/h) and other 127 patients received a moderate fluid resuscitation (RL bolus 10 ml/kg in case of hypovolemia or no bolus in normovolemic patients, followed by RL 1.5 ml/kg/h.). Patients in the aggressive resuscitation group developed a significative higher fluid overload as compared to the moderate resuscitation group, with no improvement in clinical outcomes [130].

Other ongoing RCTs are mainly focused on the comparison between normal saline and other crystalloids. Farrell et al. plan to enroll 80 pediatric patients with acute pancreatitis receiving either RL or NS to assess inflammatory markers and SIRS status at 24 and 48 h (clinicaltrials.gov NCT03242473). Poropat et al. aim to enroll 276 adult patients with acute pancreatitis to receive either Plasmalyte or normal saline as an initial bolus of 10 ml/kg in the first 60 min after randomization, and then at a rate of 2 ml/kg for the next 72 h (Clinicaltrials.Gov NCT04688645). The primary endpoint of the study is the incidence of SIRS.

## Conclusions

Fluid therapy is a key treatment of patients admitted to the ICU with severe forms of acute pancreatitis. A broadly accepted early and aggressive fluid therapy has been recently questioned due to potential harm and not definitive efficacy in clinical trials. Since there is a possible risk of under-resuscitation when a fixed infusion rate is used, a more tailored approach is warranted. It should be based on a careful assessment of the patient’s volume status, with enhanced volume expansion in the first hours of admission for the most severe cases. Crystalloids, and in particular RL, are the fluids of choice, with a suggested initial fluid rate usually ranging between 5–10 ml/kg/h. Then, if at any time during the first 24 h resuscitation goals are met, it is reasonable to reduce fluid rate to 2–3 ml/kg/h. Patients with SAP should be strictly monitored in the ICU, where advanced hemodynamic monitoring systems are available to guide clinicians.

## Abbreviations

AP: Acute pancreatitis
BUN: Blood urea nitrogen
Ht: Hematocrit
HR: Heart rate
ICU: Intensive care unit
IV: Intravenous
MAP: Mean arterial pressure
MOF: Multi-organ failure
NS: Normal saline
RCT: Randomized clinical trial
RL: Ringer lactate
SAP: Severe acute pancreatitis
SID: Strong ion difference
SIRS: Systemic inflammatory response syndrome
UO: Urine output
