# Correction: Intravenous fluid therapy in patients with severe acute pancreatitis admitted to the intensive care unit: a narrative review

**DOI:** 10.1186/s13613-023-01149-2

**Published:** 2023-06-14

**Authors:** Andrea Crosignani, Stefano Spina, Francesco Marrazzo, Stefania Cimbanassi, Manu L. N. G. Malbrain, Niels Van Regenmortel, Roberto Fumagalli, Thomas Langer

**Affiliations:** 1 School of Medicine and Surgery, University of Milan-Bicocca, Milan, Italy; 2 Department of Anaesthesia and Critical Care, ASST Grande Ospedale Metropolitano Niguarda, Milan, Italy; 3 General Surgery and Trauma Team, ASST Grande Ospedale Metropolitano Niguarda, Milan, Italy; 4 Department of Pathophysiology and Transplantation, University of Milan, Milan, Italy; 5 First Department of Anaesthesia and Intensive Therapy, Medical University of Lublin, Lublin, Poland; 6 International Fluid Academy, Lovenjoel, Belgium; 7 Department of Intensive Care Medicine, Antwerp University Hospital, Antwerp, Belgium; 8 Department of Intensive Care Medicine, Ziekenhuis Netwerk Antwerpen Campus Stuivenberg, Antwerp, Belgium

**Correction: Annals of Intensive Care (2022) 12:98** 10.1186/s13613-022-01072-y

In the original publication of the article [1], the author name Niels Van Regenmortel was incorrectly written as Niels Van Regenemortel. The original article has been corrected.
